# Patterns of Care and Survival of Metastatic Metaplastic Breast Cancer Patients

**DOI:** 10.7759/cureus.10339

**Published:** 2020-09-09

**Authors:** Irini Youssef, Anna Lee, Sparsha Kukunoor, Evelyn Taiwo, Carol A Luhrs, David Schreiber

**Affiliations:** 1 Medical Oncology, State University of New York (SUNY) Downstate Medical Center, Brooklyn, USA; 2 Radiation Oncology, The University of Texas MD Anderson Cancer Center, Houston, USA; 3 Hematology and Oncology, New York-Presbyterian Brooklyn Methodist Hospital, Brooklyn, USA; 4 Medical Oncology, State University of New York (SUNY) Downstate Medical Center/New York Harbor Healthcare System, Brooklyn, USA; 5 Radiation Oncology, Summit Medical Group, Berkeley Heights, USA

**Keywords:** metasatic metaplastic breast cancer, chemotherapy, radiation, survival

## Abstract

Background: Metaplastic breast cancer (MBC) is a rare, aggressive variant of breast cancer, usually triple negative disease and chemotherapy refractory. Despite this, the standard of care remains the same as invasive ductal breast cancer. We sought to analyze patterns of care and outcomes among patients with metastatic MBC.

Methods: Patients over 18 years diagnosed with metastatic MBC from 2004-2015 were identified in the National Cancer Database (NCDB). Clinical and demographic details were compared between two groups (chemotherapy vs no chemotherapy). Logistic regression was performed to assess for predictors of receiving chemotherapy. The Kaplan-Meier method was used to assess overall survival (OS) and Cox regression analysis was used to assess the impact of covariates on OS.

Results: There were 7,580 patients with MBC of which 417 (5.5%) presented with metastatic disease. Median age was 65 years (interquartile range (IQR) 54-76) and median follow up for living patients was 48 months (IQR 31-77). One hundred and fifty-six (37.4%) patients received chemotherapy. On multivariable logistic regression analyses, treatment at an academic facility was associated with an increased likelihood of receiving chemotherapy (OR 3.14, 95% CI 1.95-5.03, p<0.001) while age ≥65 years (OR 0.54, 95% CI 0.34-0.86, p=0.009) and receipt of hormonal therapy (OR 0.35, 95% CI 0.15-0.85, p=0.021) were associated with a decreased likelihood of receiving chemotherapy. On multivariable Cox regression analysis, higher Charlson-Deyo score (hazard ratio (HR) 1.35-1.78, p<0.05) was associated with worse survival while receipt of chemotherapy (HR 0.76, 95% CI 0.59-0.99, p=0.041) and having insurance (HR 0.34-0.47, p<0.05) were associated with improved survival. Patients who received chemotherapy had improved median (twelve versus eight months), one-year (51% versus 38%), and two-year (35% versus 21%) OS, as compared to those who did not receive chemotherapy (p=0.006).

Conclusions: In this study of MBC patients, there was a survival benefit with palliative chemotherapy in the setting of metastatic disease. As expected, treatment was most often given to younger patients.

## Introduction

Metaplastic breast cancer (MBC) is an extremely rare and aggressive malignancy, comprising less than 1% of mammary carcinomas diagnosed annually [1-3]. Relative to patients with invasive ductal carcinoma (IDC) and triple negative IDC, females with MBC are more likely to present at an older age, with larger tumor size, more advanced disease stage, more triple negative disease, and higher histologic grade [4,5]. Lymphatic spread of this tumor is rare, with metastatic disease most commonly occurring in the lung and bones [6]. MBC patients experience more disease recurrence and worse overall survival (OS) than stage-matched patients with IDC and triple-negative breast cancer [4-6]. Optimal treatment strategies for MBC are unknown, although largely paralleling those of IDC, despite accumulating evidence that MBC is a distinct clinical entity from IDC [3]. Data regarding the utility of chemotherapy in this largely aggressive and commonly metastatic disease is lacking. The purpose of this study was to analyze patterns of care and outcomes among patients with metastatic MBC receiving chemotherapy.

## Materials and methods

The National Cancer Database (NCDB) is a joint project of the American Cancer Society and the Commission on Cancer of the American College of Surgeons. It is estimated that 70% of all diagnosed malignancies in the United States are captured by facilities participating in this registry and are reported to the NCDB. The Commission on cancer’s NCDB and the hospitals participating in the NCDB are the source of the de-identified data used in this study. However, they have not verified and are not responsible for the statistical validity or conclusions derived by the authors of this study. Exemption was obtained from the Research and Development Committee of the Veterans Affairs-New York Harbor Healthcare System prior to the initiation of the study.

**Figure 1:**
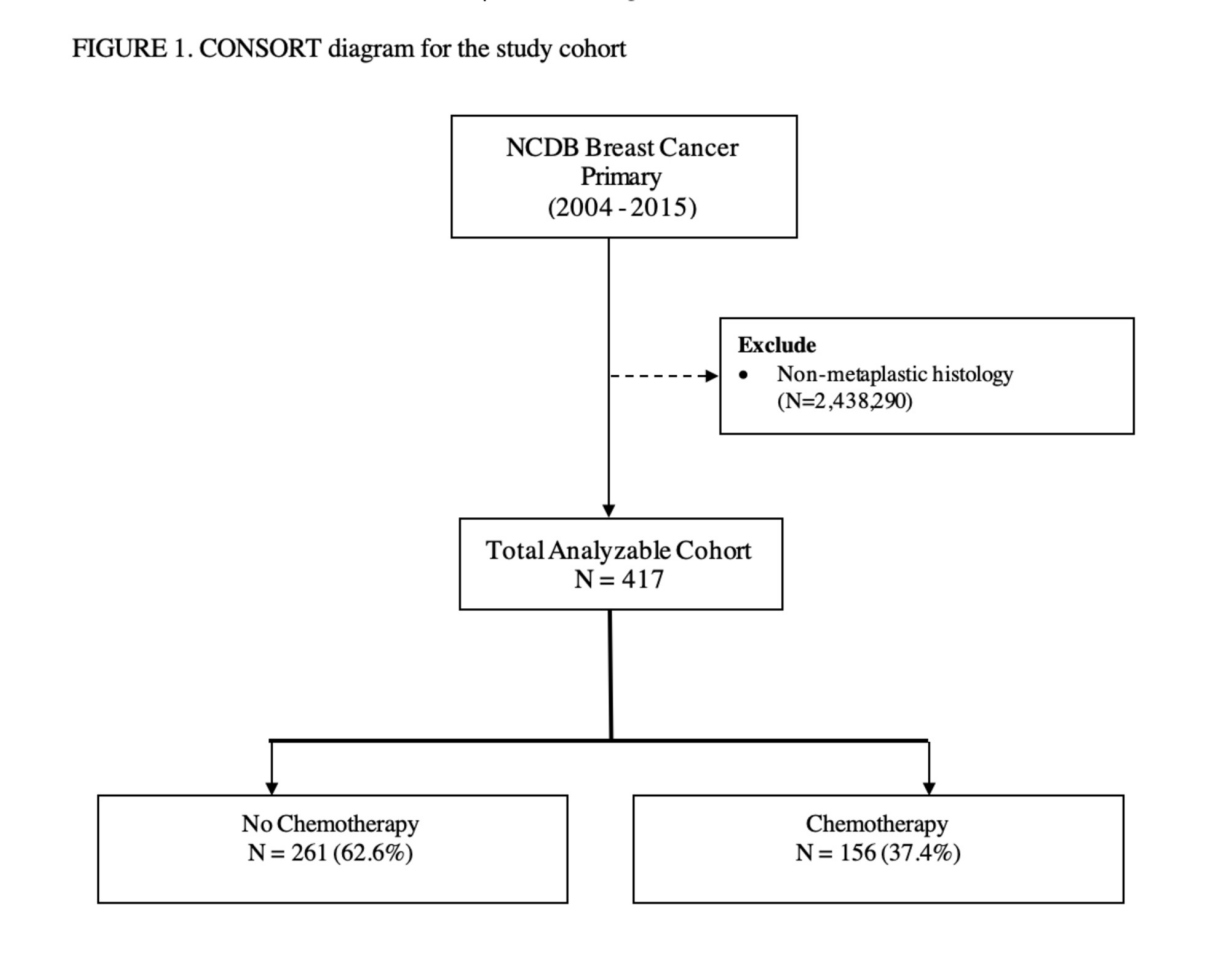
Consort diagram for study cohort

We identified 7,580 patients (0.3% of total breast cancer patients) at least 18 years of age, who were diagnosed with MBC from 2004-2015. Those who had non-metastatic disease were excluded leaving 417 patients in the final analysis (Figure 1).

Data regarding surgical, radiotherapy, hormonal, and chemotherapy treatments were collected, as well as demographic information. 

Clinical and patient-related factors were compared between patients who received chemotherapy and those who did not. The factors analyzed included age (<65, ≥65 years), race (White, Black, and Other), Charlson-Deyo score (0, 1, ≥2), estrogen receptor (ER) status (ER-, ER+, Unknown), progesterone receptor (PR) status (PR-, PR+, Unknown), HER2Neu status (HER2Neu-, HER2Neu+, Unknown), T-stage (T1, T2, T3, T4), N-stage (N0, N1, N2, N3), site of metastasis (bone, brain, liver, lung), facility type (non-academic, academic), region (Northwest, Midwest, South, West), median income quartile (<$38,000, $38,000-$47,999, $48,000-$62,999, ≥$63,000), insurance type (none, private, Medicaid, Medicare, other/unknown), years of diagnosis (2004-2007, 2008-2011, 2012-2015), receipt of hormonal therapy (no, yes) and receipt of chemotherapy (no, yes). The Charlson-Deyo comorbidity index is a method of predicting mortality by classifying comorbidities on a weighted scale.

Frequency distributions between the categorical variables were compared using the Chi-square test. Univariate and multivariate logistic regression analysis was performed to determine factors predicting receipt of chemotherapy. OS data was obtained and Kaplan-Meier curves were developed and compared via the log-rank test stratifying the groups by stage and treatment. Univariate and Cox proportional hazards regression models were performed to assess for covariates that had an impact on survival. Variables with a p-value ≤0.10 were included in the multivariate model. All analyses were conducted with SPSS 21.0 (IBM Inc, Armonk, New York). All tests were two-sided with a threshold of p<0.05 for significance. 

## Results

There were 7,580 patients with MBC of whom 417 (5.5%) presented with metastatic disease. Median age was 65 years (interquartile range (IQR) 54-76) and median follow up for living patients was 48 months (IQR 31-77). 156 (37.4%) patients received chemotherapy and 261 (62.6%) did not. Patients who did and did not receive chemotherapy were significantly different with respect to distribution of age, Charlson-Deyo comorbidity index, receipt of palliative surgery or hormonal therapy and facility type. The two groups were similar with respect to race, ER status, PR status, HER2Neu status, T-stage, N-stage, site of metastasis, receipt of palliative radiation therapy, region, insurance type, median income quartile and year of diagnosis. Further details can be found in Table 1.

**Table 1:** Patient characteristics and comparison between those receiving chemotherapy versus observation ER: estrogen receptor PR: progesterone receptor

Table 1. Patient characteristics and comparison between those receiving chemotherapy versus observation			
	No Chemo N = 261 (62.6%)	Chemo N = 156 (37.4%)	p-value
Age			<0.001
<65	111 (53.9%)	95 (46.1%)	
≥65	150 (71.1%)	61 (28.9%)	
Race			0.136
White	202 (65.2%)	108 (34.8%)	
Black	50 (53.8%)	43 (46.2%)	
Other	9 (64.3%)	5 (35.7%)	
Charlson-Deyo			0.038
0	185 (59.1%)	128 (40.9%)	
1	58 (72.5%)	22 (27.5%)	
≥2	18 (75.0%)	6 (25.0%)	
ER Status			0.526
ER-	186 (61.0%)	119 (39.0%)	
ER+	48 (67.6%)	23 (32.4%)	
Unknown	27 (56.9%)	14 (34.1%)	
PR Status			0.294
PR-	204 (62.6%)	122 (37.4%)	
PR+	7 (46.7%)	8 (53.3%)	
Unknown	147 (65.0%)	79 (35.0%)	
HER2Neu Status			0.294
HER2Neu-	107 (60.8%)	69 (39.2%)	
HER2Neu+	7 (46.7%)	8 (53.3%)	
Unknown	147 (65.0%)	79 (35.0%)	
T-stage			0.867
T1	13 (61.9%)	8 (38.1%)	
T2	40 (58.8%)	28 (41.2%)	
T3	53 (63.1%)	31 (36.9%)	
T4	90 (57.7%)	66 (42.3%)	
N-stage			0.183
N0	86 (64.2%)	48 (35.8%)	
N1	52 (51.5%)	49 (48.5%)	
N2	29 (64.4%)	16 (35.6%)	
N3	17 (53.1%)	15 (46.9%)	
Site of Metastasis			
Bone	56 (57.1%)	42 (42.9%)	0.521
Brain	17 (70.8%)	7 (29.2%)	0.507
Liver	26 (52.0%)	24 (48.0%)	0.278
Lung	87 (63.5%)	50 (36.5%)	0.652
Hormonal Therapy			0.007
No	217 (60.8%)	140 (39.2%)	
Yes	35 (81.4%)	8 (18.6%)	
Facility type			<0.001
Non-academic	191 (73.5%)	69 (26.5%)	
Academic	66 (46.2%)	77 (53.8%)	
Region			0.199
Northeast	48 (59.3%)	33 (40.7%)	
Midwest	68 (64.2%)	38 (35.8%)	
South	93 (61.2%)	59 (38.8%)	
West	48 (75.0%)	16 (25.0%)	
Insurance			0.053
Not insured	7 (53.8%)	6 (46.2%)	
Private insurance	98 (61.6%)	61 (38.4%)	
Medicaid	23 (47.9%)	25 (52.1%)	
Medicare	128 (68.8%)	58 (31.2%)	
Other/unknown	5 (45.5%)	6 (54.5%)	
Income			0.767
< $38,000	41 (59.4%)	28 (40.6%)	
$38,000-$47,999	60 (60.0%)	40 (40.0%)	
$48,000-$62,999	68 (64.2%)	38 (35.8%)	
≥$63,000	87 (65.4%)	46 (34.6%)	
Year of diagnosis			0.446
2004-2007	56 (62.9%)	33 (37.1%)	
2008-2011	93 (66.4%)	47 (33.6%)	
2012-2015	112 (59.6%)	76 (40.4%)	

On univariable logistic regression analysis, Black race (OR 1.61, 95% CI 1.01-2.57, p=0.047) and treatment at an academic center were associated with an increased likelihood of receiving chemotherapy (OR 3.23, 95% CI 2.10-4.96, p<0.001) while age ≥65 years (OR 0.48, 95% CI 0.32-0.71, p<0.001), Charlson-Deyo comorbidity index of 1 (OR 0.55, 95% CI 0.32-0.94, p=0.029), receipt of hormonal therapy (OR 0.35, 95% CI 0.16-0.79, p=0.011), treatment in the Western US (OR 0.49, 95% CI 0.24-1.00, p=0.048) were associated with a decreased likelihood of receiving chemotherapy. On multivariable logistic regression analyses, treatment at an academic facility was still associated with an increased likelihood of receiving chemotherapy (OR 3.14, 95% CI 1.95-5.03, p<0.001) while age ≥65 years (OR 0.54, 95% CI 0.34-0.86, p=0.009) and receipt of hormonal therapy (OR 0.35, 95% CI 0.15-0.85, p=0.021) were associated with a decreased likelihood of receiving chemotherapy. Further details can be found in Table 2.

**Table 2:** Univariable and multivariable logistic regression for chemo usage OR: odds ratio, CI: confidence interval

Table 2. Univariable and multivariable logistic regression for chemo usage				
	Univariable		Multivariable	
	OR (95% CI)	p-value	OR (95% CI)	p-value
Age				
<65	1		1	
≥65	0.48 (0.32-0.71)	<0.001	0.54 (0.34-0.86)	0.009
Race				
White	1		1	
Black	1.61 (1.01-2.57)	0.047	1.39 (0.80-2.41)	0.244
Other	1.04 (0.34-3.18)	0.946	0.90 (0.24-3.38)	0.877
Charlson-Deyo				
0	1		1	
1	0.55 (0.32-0.94)	0.029	0.60 (0.32-1.10)	0.095
≥2	0.48 (0.19-1.25)	0.132	0.58 (0.32-1.10)	0.324
Palliative Surgery				
No	1		1	
Yes	0.67 (0.45-1.01)	0.053	0.71 (0.45-1.12)	0.142
Palliative Radiation Therapy				
No	1		-	-
Yes	0.95 (0.62-1.44)	0.799	-	-
Hormonal Therapy				
No	1		1	
Yes	0.35 (0.16-0.79)	0.011	0.35 (0.15-0.85)	0.021
Facility type				
Non-academic	1		1	
Academic	3.23 (2.10-4.96)	<0.001	3.14 (1.95-5.03)	<0.001
Region				
Northeast	1		1	
Midwest	0.81 (0.45-1.47)	0.495	1.08 (0.55-2.10)	0.829
South	0.92 (0.53-1.60)	0.775	1.25 (0.66-2.35)	0.494
West	0.49 (0.24-1.00)	0.048	0.59 (0.26-1.32)	0.198
Insurance				
Not insured	1		-	-
Private insurance	0.73 (0.23-2.26)	0.581	-	-
Medicaid	1.27 (0.37-4.33)	0.705	-	-
Medicare	0.53 (0.17-1.64)	0.270	-	-
Other/unknown	1.40 (0.28-7.02)	0.682	-	-
Income				
< $38,000	1		-	-
$38,000-$47,999	0.98 (0.52-1.82)	0.940	-	-
$48,000-$62,999	0.82 (0.44-1.53)	0.528	-	-
≥$63,000	0.77 (0.43-1.41)	0.402	-	-
Year of diagnosis				
2004-2007	1		-	-
2008-2011	0.86 (0.49-1.49)	0.588	-	-
2012-2015	1.15 (0.69-1.94)	0.595	-	-

On univariate Cox regression analysis, age ≥65 years (HR 1.48, 95% CI 1.17-1.88, p=0.001) and Charlson-Deyo score ≥1 (HR 1.50-2.12, p<0.05) were associated with worse survival while receipt of chemotherapy (HR 0.71, 95% CI 0.55-0.91, p=0.006) was associated with improved survival. Receipt of hormonal therapy (HR 0.77, 95% CI 0.51-1.15, p=0.201) was not significantly associated with survival. On multivariable Cox regression analysis, higher Charlson-Deyo score ≥2 (HR 1.78, 95% CI 0.99-1.83, p=0.019) was associated with worse survival. In contrast, having private insurance (HR 0.47, CI .24-.94, p<0.05) or Medicaid (HR 0.34 95% CI 0.16-0.73, p<.006) and receipt of chemotherapy (HR 0.76, 95% CI 0.59-0.99, p=0.041) were associated with improved survival. Further details can be found in Table 3. 

**Table 3:** Univariable and multivariable Cox regression for overall survival ER: estrogen receptor HR: hazard ratio, CI: confidence interval

Table 3. Univariable and multivariable Cox regression for overall survival				
	Univariable		Multivariable	
	HR (95% CI)	p-value	HR (95% CI)	p-value
Age				
<65	1		1	
≥65	1.48 (1.17-1.88)	0.001	1.31 (0.86-2.01)	0.209
Race				
White	1		-	-
Black	0.98 (0.74-1.30)	0.900	-	-
Other	0.51 (0.23-1.14)	0.101	-	-
Charlson-Deyo				
0	1		1	
1	1.50 (1.11-2.02)	0.008	1.35 (0.99-1.83)	0.059
≥2	2.12 (1.33-3.38)	0.002	1.78 (0.99-1.83)	0.019
Hormone Receptor Status				
ER-	1		-	-
ER+	0.851 (0.62-1.18)	0.329	-	-
Unknown	1.12 (0.0.77-1.64)	0.553	-	-
Hormonal Therapy				
No	1		-	-
Yes	0.77 (0.51-1.15)	0.201	-	-
Chemotherapy				
No	1		1	
Yes	0.71 (0.55-0.91)	0.006	0.76 (0.59-0.99)	0.041
Palliative Surgery				
No	1		-	-
Yes	0.59 (0.30-1.13)	0.109	-	-
Palliative Radiation Therapy				
No	1		-	-
Yes	1.50 (0.82-2.74)	0.193	-	-
Facility type				
Non-academic	1		-	-
Academic	0.98 (0.76-1.26)	0.861	-	-
Region				
Northeast	1		-	-
Midwest	1.21 (0.83-1.74)	0.321	-	-
South	1.09 (0.77-1.56)	0.625	-	-
West	1.04 (0.68-1.58)	0.867	-	-
Insurance				
Not insured	1		1	
Private insurance	0.56 (0.28-1.10)	0.091	0.47 (0.24-0.94)	0.032
Medicaid	0.37 (0.17-0.79)	0.011	0.34 (0.16-0.73)	0.006
Medicare	0.74 (0.37-1.45)	0.377	0.46 (0.21-1.01)	0.052
Other/unknown	0.41 (0.14-1.14)	0.088	0.37 (0.13-1.04)	0.060
Income				
< $38,000	1		-	-
$38,000-$47,999	0.93 (0.64-1.35)	0.695	-	-
$48,000-$62,999	1.24 (0.85-1.80(	0.270	-	-
³$63,000	0.93 (0.64-1.35)	0.710	-	-
Year of diagnosis				
2004-2007	1		-	-
2008-2011	1.04 (0.79-1.38)	0.772	-	-
2012-2015	0.93 (0.67-1.29)	0.657	-	-

**Figure 2:**
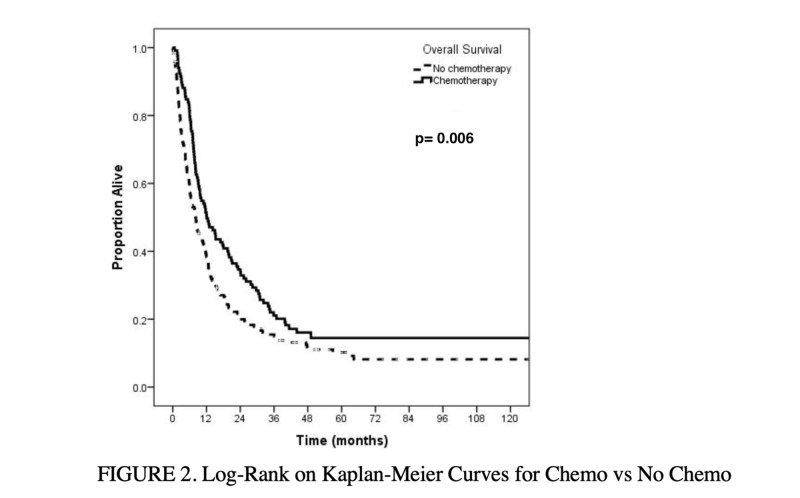
Overall survival: chemotherapy versus no chemotherapy P value bolded to highlight statistical significance

On unadjusted Kaplan-Meier analysis, median survival for those who received chemotherapy was 12 months compared to eight months for those who did not receive chemotherapy (p=0.006) (Figure 2). Additionally, patients who received chemotherapy had improved median (twelve versus eight months), one-year (51% versus 38%), and two-year (35% versus 21%) overall survival as compared to those who did not receive chemotherapy (p=0.006). 

## Discussion

MBC is a rare and histologically diverse subtype of breast cancer, first described by Huvos et al. in 1974 [7]. Histologically, it is a poorly differentiated heterogeneous tumor containing ductal carcinoma cells admixed with areas of spindle, squamous, chondroid, or osseous elements [8]. It comprises less than 1% of all breast cancers, and presents at a median age of 48-59 years [8]. It less frequently presents with nodal metastases than IDC, despite its larger size at presentation, and is also more likely to present initially with higher stage or metastatic disease [9]. A NCDB analysis demonstrated MBC patients were more likely to present with stage III disease (10.6% vs 8.4%) and stage IV disease (4.6% vs. 3.4%) and have triple negative disease (11.3% vs. 74.1%, OR, 22.4, P = .001), when compared to IDC [9]. Patients with MBC also tend to have more local and distant (often bone and lung) metastases than IDC, and worse prognosis than IDC and triple-negative breast cancer [6]. Breast-conserving surgery is utilized less frequently in MBC, and these patients are more likely to receive chemotherapy than patients with IDC and triple-negative IDC [9].

Despite its extremely aggressive nature, there are no current management guidelines for MBC due to both its rarity, and its incompletely understood natural history. To date, there have been no randomized control trials evaluating the role of chemotherapy in the management of MBC in patients with metastatic disease and cohort studies focus largely on localized disease. To our knowledge, the current study is the largest retrospective study evaluating the impact of chemotherapy on survival outcomes in patients with metastatic MBC. Of the 417 patients with metastatic MBC included in this study, we found that 37.4% received chemotherapy. Studies report that 33%-86% of MBC patients receive chemotherapy, and are two times as likely to receive chemotherapy as matched patients with IDC [9-11]. Most single-institution cohort studies suggest these tumors are largely chemo-resistant, which makes it difficult to draw satisfactory conclusions, and the benefits of chemotherapy have mostly been shown in non-metastatic disease [11,12]. A very limited number of small sample studies report on chemotherapy in metastatic disease. Chen et al. reported on 12 of 18 patients with metastatic MBC who received systemic therapy. The authors found that only two patients (16.7%) had a partial response, and 83.3% experienced disease progression [13]. Similarly, Rayson et al. reported one partial response in seven patients with metastatic disease who received adjuvant chemotherapy [10]. In studies with larger patient samples, treatment with chemotherapy appears promising, but with still somewhat limited value. Among 30 patients with recurrent, metastatic or de novo stage IV disease, Lee et al. reported an overall-response rate of 38.9% [14]. Another study found that lack of adjuvant chemotherapy was associated with an almost four-fold decrease in OS (HR, 3.67; 95% CI, 1.1-12.4; P = .036) on univariate analysis [15]. In our study, we found a statistically significant difference in both one and two-year OS between patients who received chemotherapy versus those who did not (one-year OS 50.6% versus 37.8%, and, two-year OS 34.6 versus 20.5%, respectively, p=0.006). Additionally, on multivariable analysis, the use of chemotherapy was associated with improved OS (HR .76 (.59-.99). Factors related to specific chemotherapy regimens and the presence of lymph node positive disease have been found to impact treatment outcomes [11,12].

As the NCDB does not report on factors relating to the choice of chemotherapy versus no chemotherapy for patients, it is difficult to draw conclusions as to why some patients were chosen for a certain treatment compared to others. On logistic regression, we found that a Charlson-Deyo score of 0 was associated with the receipt of chemotherapy on univariate analysis. Additionally, patients younger than 65 years old were more likely to receive chemotherapy on multivariate analysis, as 46% of patients younger than 65 years received chemotherapy compared to only 29% of patients 65 years and older. It appears that age may be a contributing factor on patient selection for chemotherapy, although many studies have shown that age is not significantly associated with survival outcomes [11,12,16,17]. This may be due to the higher likelihood of chemotherapy toxicity in the elderly, although that is also difficult to speculate as specific treatment regimens and their toxicities have not been studied in trials and there are no other studies analyzing patterns of care in metastatic MBC.

MBC is known to be predominantly triple negative (ER, PR, HER2 receptor negative) [12,18]. This biological difference represents obvious implications in treatment, and even triple-negative ductal carcinoma confers a less poor outcome compared to triple negative metaplastic patients [19,20]. However, the presence of hormone receptor positivity in MBC patients has not been shown to impact prognosis [12,18,21]. In one study on 54 patients with MBC, the use of hormone replacement therapy (HRT) did not provide better outcomes in MBC patients who had hormone-receptor positive tumors (HRT+ vs. HRT-, for progression-free survival (PFS), 70 ± 6% vs. 66 ± 16%, P = 0.34, and for OS, 83 ± 4% vs. 77 ± 14%, P = 0.42) [22]. Rayson et al. found no response in four ER/PR positive patients treated with tamoxifen at the time of relapse [10]. Our study found hormone negative disease was significantly associated with treatment with chemotherapy, although we did not find a significant survival difference between hormone receptor status or in patients who received hormone therapy. Due to the high incidence of triple-negative receptor status in MBC, hormone targeted treatment is unlikely to influence survival.

There are limitations to this study. While data reporting to the NCDB is highly standardized, there may still be variations in data abstraction, particularly with non-academic community-based oncology practices. Other limitations include lack of coding regarding the chemotherapy agent used and the inability to assess tumor burden. We are also unable to account for toxicities associated with chemotherapy and this may have impacted which patients were able to receive chemotherapy. Finally, it is uncertain why certain patients were selected for more aggressive therapies over others. As the NCDB is unable to account for why certain patients received chemotherapy versus others, the improved survival associated with chemotherapy is hypothesis generating and would need to be confirmed in more comprehensive prospective studies. The strengths of this study include the large patient sample from a highly representative database.

## Conclusions

Although highly aggressive, MBC is a relatively rare disease, contributing to the lack of randomized control trials. The rarity of the disease has led to small sample sizes in several retrospective studies with conflicting results on the benefits of chemotherapy, radiation therapy, and surgery. In this large and highly representative NCDB study, we found the use of chemotherapy to be associated with improved survival. However, randomized control trials are needed to further evaluate the optimal treatment for patients with this disease.
